# Impact of the hypoxic phenotype on the uptake and efflux of nanoparticles by human breast cancer cells

**DOI:** 10.1038/s41598-018-30517-3

**Published:** 2018-08-17

**Authors:** William J. Brownlee, F. Philipp Seib

**Affiliations:** 1 Strathclyde Institute of Pharmacy and Biomedical Sciences, University of Strathclyde, 161 Cathedral Street, Glasgow, G4 0RE UK; 2 Leibniz Institute of Polymer Research Dresden, Max Bergmann Center of Biomaterials Dresden, Hohe Strasse 6, 01069 Dresden, Germany

## Abstract

Breast cancer cells adapt to the hypoxic tumoral environment by undergoing changes in metabolism, cell signalling, endo-lysosomal receptor uptake and recycling. The resulting hypoxic cell phenotype has the potential to undermine the therapeutic efficacy of nanomedicines designed for endocytic uptake and specific intracellular trafficking. The aim of this study was to examine the impact of hypoxia and simulated reperfusion on the *in vitro* uptake and release of nanomedicines by human breast cancer cells. Cells were exposed to a hypoxic preconditioning treatment in 1% oxygen for 6 and 24 hours to induce temporal changes in the hypoxic circuit (e.g. HIF-1α expression). The preconditioned cells were then dosed with nanoparticles for 45 or 180 minutes emulating nanomedicine access following tumor reperfusion. Hypoxic preconditioning significantly increased nanoparticle retention by up to 10% when compared to normoxic cultures, with the greatest relative difference between normoxic and hypoxic cultures occurring with a 45 minute dosing interval. Exocytosis studies indicated that the preconditioned cells had a significantly increased nanoparticle efflux (up to 9%) when compared to normoxic cells. Overall, we were able to show that hypoxic preconditioning regulates both the endocytosis and exocytosis of nanomedicines in human breast cancer cells.

## Introduction

Cancer nanomedicines are typically macromolecular drug delivery systems in the nanometer size range that are developed to reduce systemic toxicity but that also have the potential to exploit key features of solid tumor pathophysiology namely, leaky blood vessels and reduced lymphatic drainage to enhance passive tumor accumulation^1,2^. Despite decades of research^2,3^, only a few anticancer nanomedicines are currently in routine clinical use; for example, Abraxane® (nanoparticle albumin-bound paclitaxel), Myocet® (liposomal doxorubicin), Doxil® (PEGylated liposomal doxorubicin), marketed as Caelyx® within Europe, Onivyde® (PEGylated liposomal irinotecan) and Daunoxome® (liposomal daunorubicin) are approved for treatment of solid tumors^4^. Specifically, Abraxane®^5^, Caelyx®^6^ and Myocet®^7^ are licensed for the treatment of advanced metastatic breast cancer no longer responsive to estrogen, progesterone and ERBB2 (Her2/neu) targeted therapies. The primary motivation for the development of these nanomedicine formulations has been the improvement in side effect profiles (e.g. reduction in doxorubicin-associated cardio toxicity) enabling the use of these cytotoxic drugs in heavily pre-treated patients^6^. However, the overall small number of this anticancer nanomedicine arsenal generally, reflects the difficulties encountered in the successful development of anticancer nanomedicines from concept through clinical practice^8,9^.

Many anticancer nanomedicine designs currently in preclinical and clinical development exploit the leaky vasculature and reduced lymphatic drainage of solid tumors as these tumor features favour the passive accumulation of nanomedicines at the tumor sites. This phenomenon, first described in 1986 and now commonly referred to as the “enhanced permeability and retention” (EPR) effect^10^. This arises due to a number of factors, including intratumoral hypoxia. Hypoxia in turn triggers angiogenesis and neo vascularisation principally via vascular endothelial growth factor^11,12^, platelet derived growth factor β and angiopoeitin-2 (ref.^13^). The result is dysregulated and chaotic vascular growth, which commonly lacks stabilising smooth muscle cells. These abnormal blood vessels are heterogeneous but typically characterised by defective, irregular vascular endothelial cell coverage^14^. These defective endothelial cells exhibit enlarged intercellular fenestrations, which facilitate the (passive) tumorotropic transit and accumulation of nanomedicines (or macromolecules) within solid tumors (i.e. the EPR effect)^15^; acting against this trend is the raised internal tumor pressure^16^. Exploitation of the EPR effect in a clinical setting has proven difficult, and emerging evidence calls for better EPR-positive patient stratification using image-guided approaches; this has now been pioneered in advanced metastatic breast cancer patients^17^.

Both tumor vascular development and density^18^, as well as perfusion and hypoxia, are key regulators of nanomedicine distribution because nanomedicines are typically administered intravenously and must therefore successfully complete their journey from the injection site to the tumor. Intratumoral hypoxia can be intermittent or transient^19,20^, which means that the physical access of a nanomedicine to hypoxic breast cancer tumor cells may be restricted to short, transient periods of vascular reperfusion. During reperfusion, the nanomedicine must navigate physical barriers, such as the extracellular matrix and immune and cancer-associated cells (e.g., fibroblast, macrophages etc.), and must overcome physiological factors (e.g., high interstitial fluid pressure) to reach the core of solid (breast) tumors^21^.

Hypoxia within the solid tumor itself is of particular importance. Typically, survival of tumor cells under hypoxic stress requires adaptation via a series of hypoxic induction factors (HIF), principally HIF1^22,23^. These factors consist of a constitutively expressed β subunit (ARNT; aryl hydrocarbon receptor nuclear translocator) and one of three oxygen-labile α subunits (denoted 1, 2 and 3). During periods of hypoxia, HIF1α, rather than undergoing normal proteasomal degradation^24^, translocates to the nucleus, where it combines with the HIFβ subunit to act on the conserved consensus sequence 5′-(A/G) CGTG-3′^25^, the hypoxic response element, in the promoter region of over 1,000 genes^26,27^. This triggers a cascade of cellular changes, with the overall result being clinically aggressive, highly metastatic^28,29^ and treatment resistant^30,31^ tumor growth.

However, of potentially greater significance from a nanomedicine perspective is that hypoxic adaptation also alters key cellular processes, including energy metabolism^32–34^, endocytic receptor internalisation^35^, transmembrane receptor recycling, trafficking^36^ and signalling^37^. Nanomedicines designed for intracellular activation in cancer cells rely on endocytosis and correct intracellular trafficking for effective therapeutic payload delivery. The energy dependence of endocytic uptake of nanomedicines means that these hypoxia-induced changes have the potential to directly undermine fundamental nanomedicine design principals. Therefore, an inherent link exists between hypoxic status, re-oxygenation of hypoxic tumor cells and the cellular presentation and internalisation of nanomedicines. However, few if any studies have sought to rigorously quantify the impact of these biological changes upon nanomedicine uptake and retention. Given the dynamic nature of the hypoxic response and the myriad changes observed within hypoxic tumor cells, the aim of this study was to quantify, *in vitro*, the impact of hypoxia exposure, simulated reperfusion and dosing interval on nanomedicine internalisation and retention in triple negative, clinically aggressive human breast cancer cells. The MDA-MB-231 cell line was selected, because it is representative of the most difficult to treat breast cancer subtype (triple negative breast cancer)^38^, which is deficient in estrogen, progesterone and ERBB2 (Her2/neu) receptors^39^, MDA-MB-231 cells are therefore unresponsive to hormone (e.g., Tamoxifen®) or receptor based therapies (e.g., Herceptin®) which are typical used to treat other breast cancer types. Therefore to make progress with triple negative breast cancer there is the urgent need to better understand the performance of nanomedicines (e.g. nanoparticles) in the presence of hypoxia. This study quantified the uptake and efflux of nanoparticles in hypoxic conditioned MDA-MB-231 breast cancer cells and the bone metastatic subpopulation. In parallel, expression of key biological markers of the hypoxic cell stress response, including HIF1-α, was assessed.

## Results

### Monitoring the pericellular oxygen concentration

**Figure 1:**
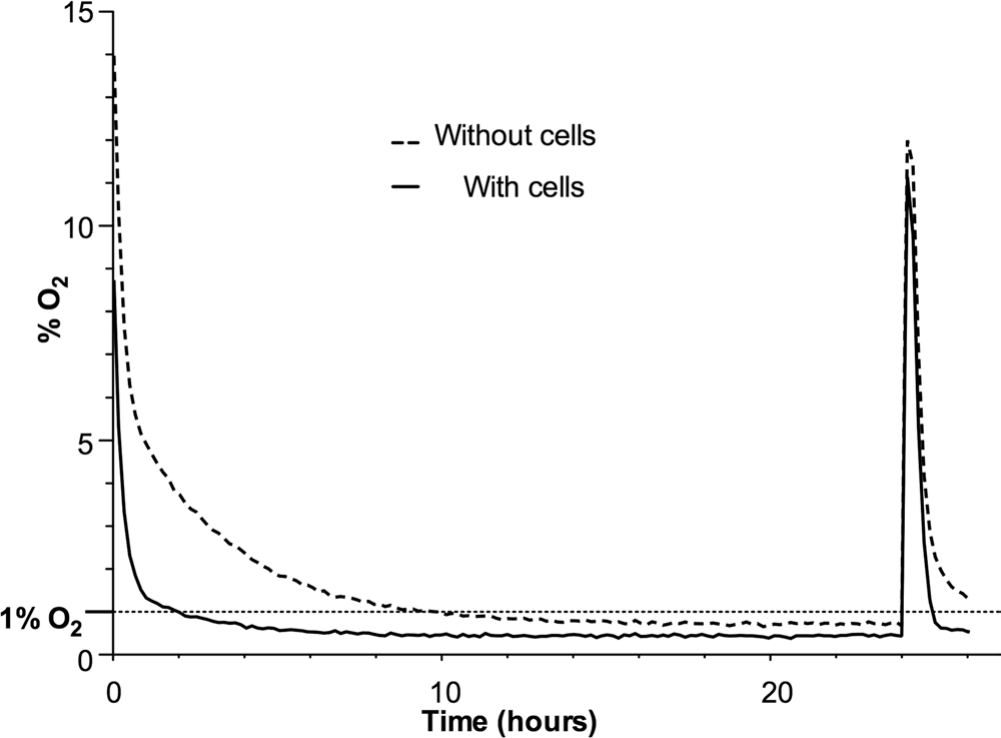
Pericellular oxygen monitoring to emulate transient intratumoral reperfusion of breast tumor. Typical mean pericellular oxygen for cell culture wells with and without MDA-MB-231 cells. Pericellular oxygen monitoring over the entire experimental time course (for magnification see Supplementary Fig. S1). Data average n = 3 (for ± SD see Supplementary Fig. S1).

Mean pericellular oxygen was measured at each time point, for wells with media only or media plus MDA-MB-231 cells. Monitoring of pericellular oxygen levels, measured within cell culture wells, allowed an evaluation of the actual oxygen levels cultures were exposed to, as opposed to the regulated 1% oxygen environment in which they were conditioned. Following 30 minutes hypoxic exposure the mean percentage pericellular oxygen level for wells containing cells was lower than those with media only (2.3% versus 6.3% respectively) (Fig. 1, Supplementary Fig. S1). Pericellular oxygen levels reached 1.33%, following 1 hour incubation, ultimately declining to a mean pericellular oxygen level of 0.44% between the 10 and 20 hour hypoxic conditioning period (Fig. 1). Exposure to atmospheric oxygen at 24 hours conditioning, resulted in transient elevation of pericellular oxygen levels, emulating the intermittent reperfusion seen with hypoxic tumors. Values returned to ≤ 1% within 50 minutes of resumption of hypoxic conditions.

### Impact of fluorescent polystyrene nanoparticles on cell viability and trafficking

**Figure 2:**
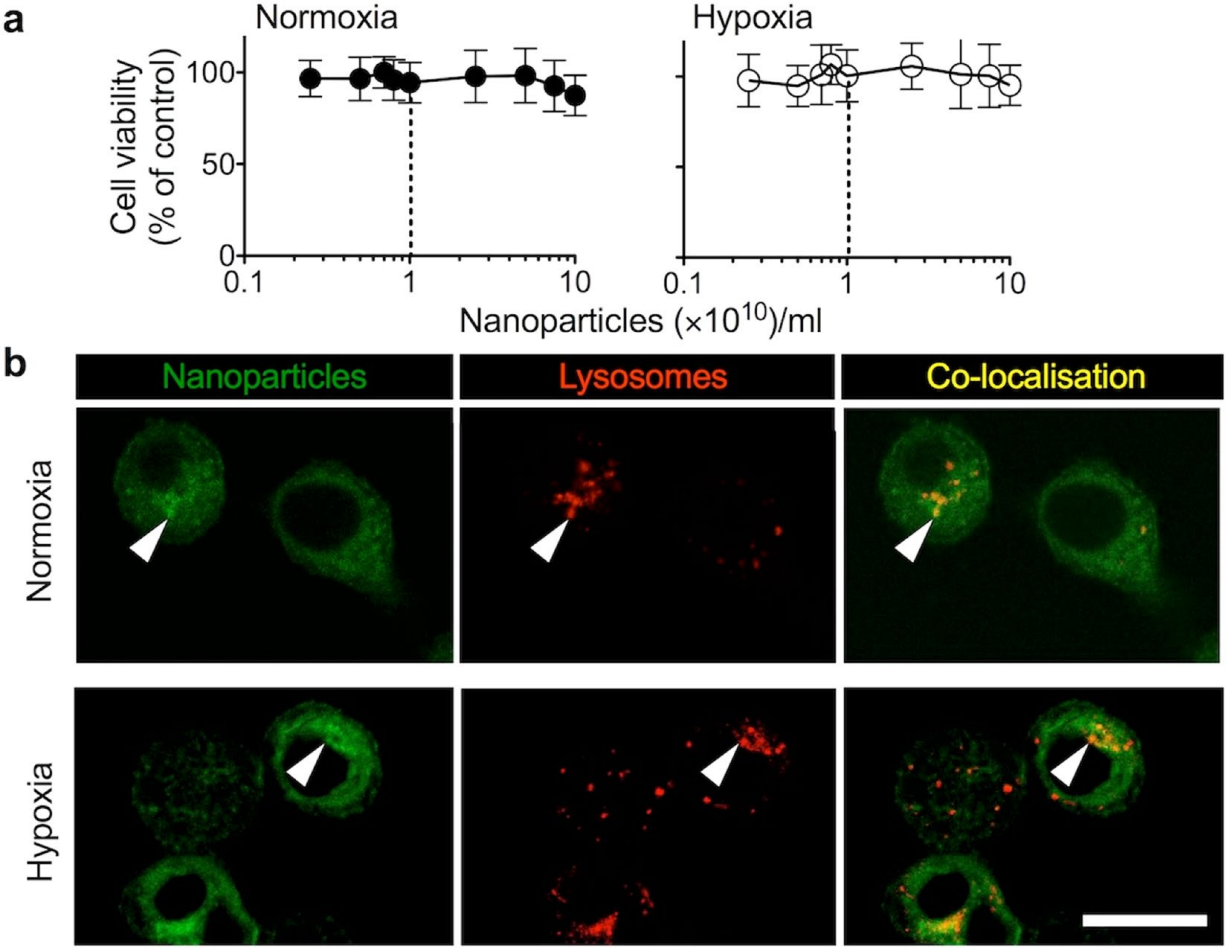
Cytotoxicity and uptake of nanoparticles in response to normoxia and hypoxia. (**a**) *In vitro* cytotoxicity of fluorescent nanoparticles in the MDA-MB-231 human breast cancer cells. Cells were dosed with fluorescent nanoparticles and subsequently cultured in either hypoxia (1% O_2_) or normoxia. At 48 hours cell viability was assessed. Dotted lines indicate the nanoparticle dose used for subsequent studies (*n* = 18 at each dosing point, from three biological replicates; ± SD). (**b**) Representative live cell confocal imaging of cells exposed for 24 hours to normoxia or hypoxia and subsequently dosed for 45 minutes with nanoparticles (green). Acidic vesicles were stained using LysoTracker Red. Arrows show nanoparticle co-localisation in acidic vesicles. Scale bar 20 μm.

The potential confounding effects from reduced cell viability due to nanoparticles (diameter 43.4 nm ± 4.4) were excluded by assessing cell viability first. We selected this particle size because clinically used nanomedicines are typically within the 10 to 100 nm size range^1,2^. Furthermore, particles with a nominal diameter of 50 nm have no restrictions with respect to uptake routes into cells, which is encountered at larger particle sizes (e.g. >100 nm limited caveola uptake)^2^. Furthermore, polystyrene nanoparticles were selected to minimize any confounding effects (e.g. alternations of plasma membrane/endocytic membrane compositions)^2^. Cell viability over a 48 hour period was similar under either hypoxic or normoxic incubation conditions following exposure to the range of nanoparticle concentrations (Fig. 2a); no biological significant reduction of cell viability was observed (IC_50_ > 10^11^ nanoparticles/ml). Dual wavelength confocal imaging of live MDA-MB-231 cells revealed co-localisation of the fluorescent nanoparticles with acidic intracellular vesicles, indicating endolysosomal uptake for both control and hypoxic cultures (Fig. 2b). The overall trafficking pattern was similar for both normoxic and hypoxic cultures following a 45 minutes and 180 minutes exposure to nanoparticles (Supplementary Fig. S2).

### Assessment of the hypoxic phenotype of MDA-MB-231 cells

**Figure 3:**
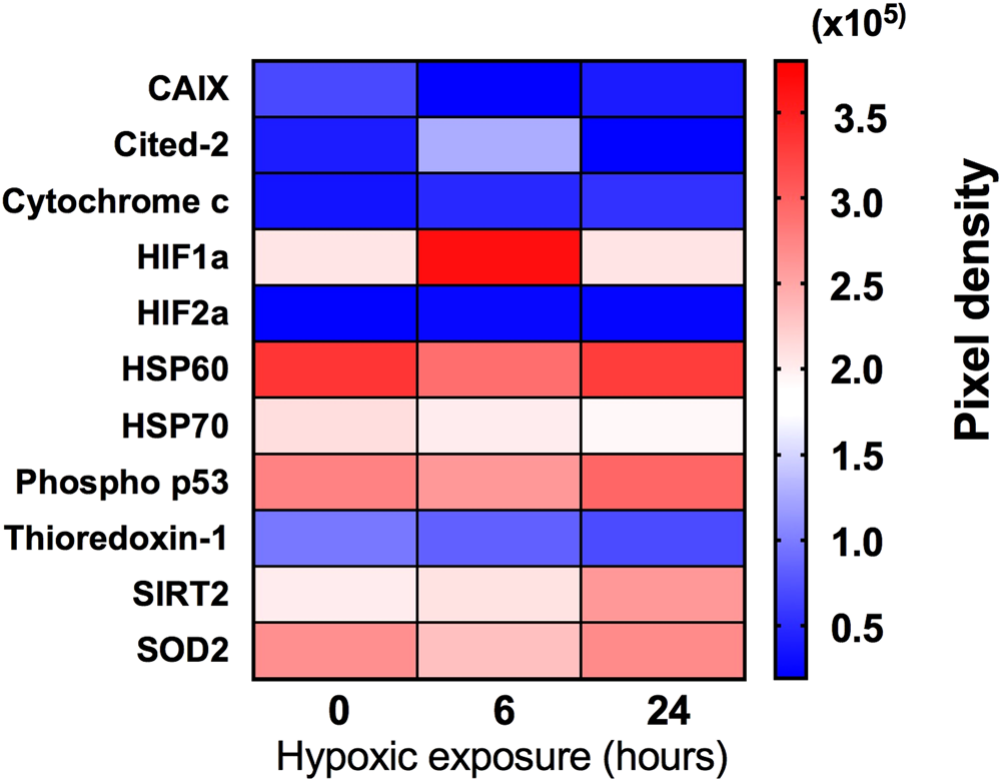
Impact of hypoxia on cell stress related proteins in human MDA-MB-231 breast cancer cells. Cells were conditioned in hypoxia (1% O_2_) for either 0, 6 or 24 hours. Relative expression of cell stress related proteins in whole cell lysates using antibody protein arrays (pooled lysates from 3 samples). Full names: *CAIX, carbonic anhydrase 9; Cited-2, CbP/p300 – interacting transactivator – 2; HIF1α, α subunit of hypoxic induction factor 1; HIF2α, α subunit of hypoxic induction factor 2; HSP60, heat shock protein 60; HSP70, heat shock protein 70; Phospho p53 (s46), phosphorylated p53; SIRT2, NAD-dependent deacetylase sirtuin-2; SOD2, mitochondrial superoxide dismutase 2*.

The phenotypic adaptation of MDA-MB-231 cells exposed to hypoxia was monitored by assessing the expression of 11 cell stress related proteins, including HIF1α, the key effector of hypoxic adaptation, after 0, 6 and 24 hours of hypoxic conditioning (Fig. 3). The protein array results from cell lysates demonstrated differentially regulated cell stress associated proteins. For example, HIF1α levels were highest after 6 hours of hypoxia but remained slightly elevated after 24 hours of hypoxia when compared to normoxic cultures. Carbonic anhydrase 9 expression showed a close to 2 fold increase from 6 hours to 24 hours (relative measured fluorescence 21,003 and 39,116, respectively). CbP/p300 – interacting transactivator -2 (Cited-2) exhibited a similar expression profile, with elevated levels following 6 hours hypoxia, whereas Thioredoxin-1 increased progressively across the 24 hours hypoxic period (Fig. 3).

HIF1α is a master regulator of the hypoxic response; therefore, the protein array results were verified by SDS PAGE and western blotting to determine the relative HIF1α expression in biological replicates (Supplementary Fig. S3a). The immunoblotting results confirmed the HIF1α expression pattern observed with the protein array (Fig. 3, Supplementary Fig. S3b); namely, the HIF1α expression levels were highest after 6 hours of hypoxic incubation (a 4.10-fold increase) and lower at 24 hours (a 1.54-fold increase), whereas the control cultures showed no substantial change in HIF1α (1.0-fold).

### Nanomedicine uptake and release by normoxic and hypoxic breast cancer cells

**Figure 4:**
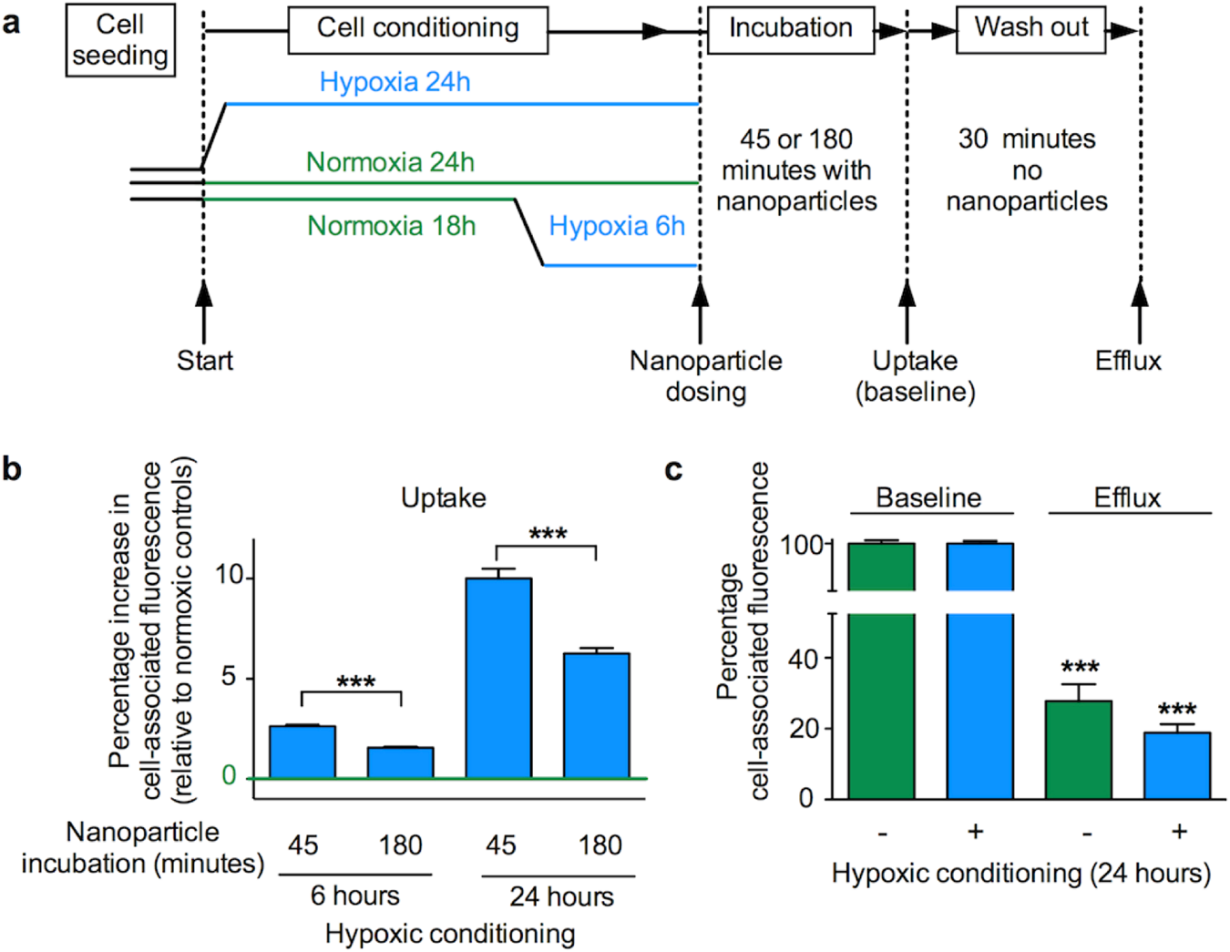
Impact of hypoxic preconditioning on the uptake (endocytosis) and recycling (exocytosis) of nanoparticles by human MDA-MB-231 breast cancer cells. (**a**) Diagram of the experimental approach. Cells were conditioned in hypoxia (1% O_2_) for either 6 or 24 hours and then dosed with nanoparticles at an effective concentration of 1 × 10^10^ nanoparticles/ml for either 45 or 180 minutes. For the recycling studies, cells were preconditioned for 24 hours in either hypoxia (1% O_2_) or normoxia. Next, cells were dosed with nanoparticles at an effective concentration of 1 × 10^10^ nanoparticles/ml for 45 minutes and analysed (baseline), or washed and allowed to exocytose for 30 minutes (exocytosis) (**b**) Cell uptake of fluorescent nanoparticles was assessed by measuring mean single cell-associated fluorescence by flow cytometry. (**c**) Exocytosis of fluorescent nanoparticles of normoxia or hypoxia preconditioned cells. For (**b**,**c**) mean single cell-associated fluorescence was measured by flow cytometry; ≥10,000 events; *n* = 15 for each dosing group and treatment period, 3 independent biological experiments ± SD.

**Figure 5:**
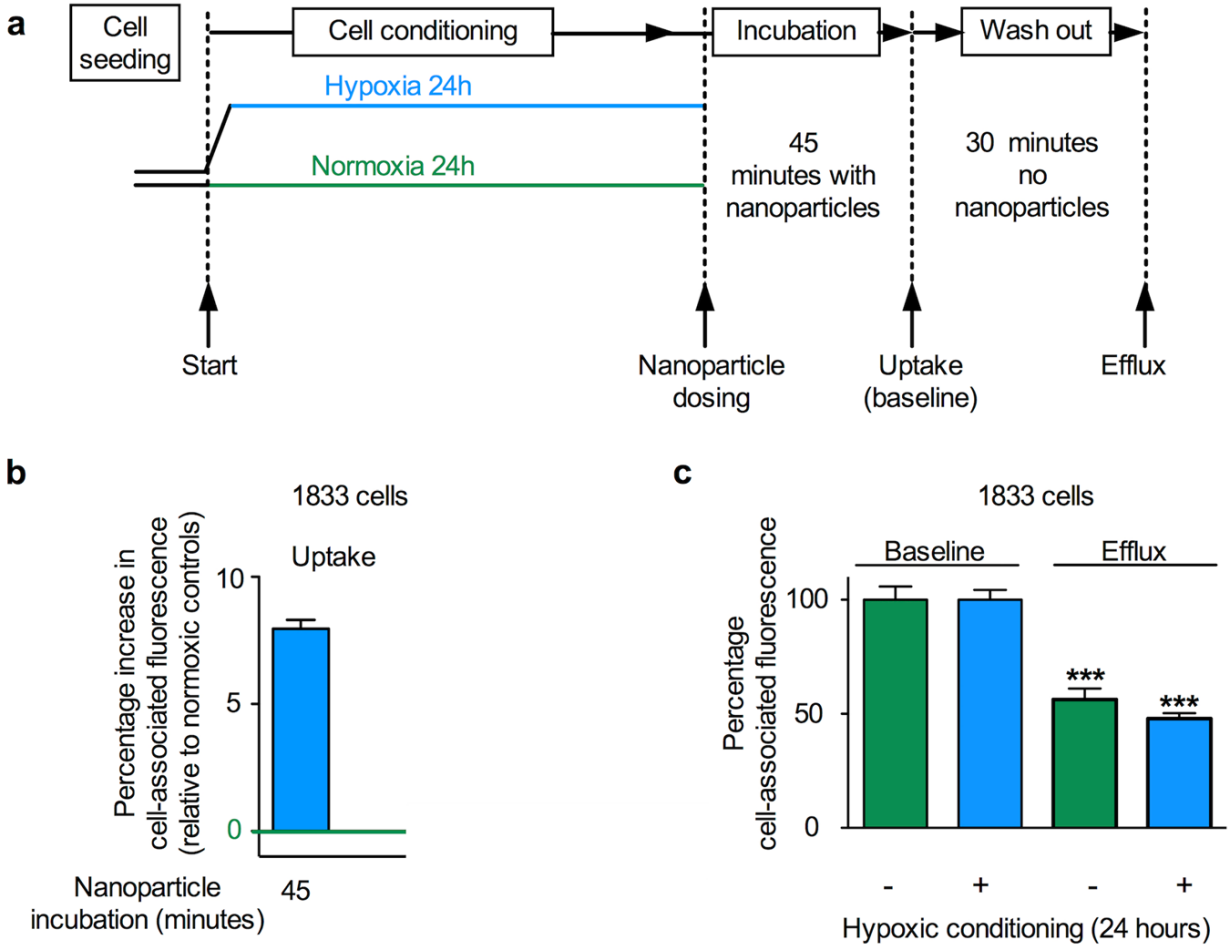
Impact of hypoxic preconditioning on the uptake (endocytosis) and recycling (exocytosis) of nanoparticles by human 1833 breast cancer cells. (**a**) Diagram of the experimental approach. Cells were conditioned in hypoxia (1% O_2_) for 24 hours and then dosed with nanoparticles at a concentration of 1 × 10^11^ nanoparticles/ml for 45 minutes. For the recycling studies, cells were preconditioned for 24 hours in either hypoxia (1% O_2_) or normoxia. Next, cells were similarly dosed for 45 minutes and analysed (baseline) or washed and allowed to exocytose for 30 minutes (exocytosis) (**b**) Cell uptake of fluorescent nanoparticles was assessed by measuring mean single cell-associated fluorescence by flow cytometry. (**c**) Exocytosis of fluorescent nanoparticles of normoxia or hypoxia preconditioned cells. Mean single cell-associated fluorescence was measured by flow cytometry, ≥10,000 events per measurement; (**b**) n = 15 from 3 independent biological experiments, (**c**) *n* = 20 for each treatment period, 2 independent biological experiments ± SD.

The relative expression of cell stress related proteins, and in particular, the differential expression of HIF1α observed after 6 or 24 hours of hypoxic conditioning led to the choice of these time points for uptake studies in MDA-MB-231 human breast cancer cells (Fig. 4a). Following the respective hypoxia conditioning, MDA-MB-231 cells were exposed to nanoparticles for either 45 or 180 minutes. When compared to the respective normoxic controls, nanoparticle uptake at 45 minutes was significantly increased in cells exposed to 6 hours of hypoxic conditioning. With the same hypoxic conditioning regime nanoparticle uptake was substantially upregulated at 180 minutes (Fig. 4). By contrast, cells conditioned for 24 hours under hypoxia showed significantly increased nanoparticle uptake at both the 45 and 180 minute dosing intervals when compared to normoxic control cultures. The largest overall upregulation of nanoparticle uptake (10.02% ± 5.36) was observed at the 45 minute dosing interval in cells conditioned under hypoxia for 24 hours (Fig. 4b). These observations were verified with the 1833 breast cancer subline (originally derived from MDA-MB-231 via *in vivo* selection^40^). 1833 cells were conditioned for 24 hours in hypoxia and dosed with nanoparticles for 45 minutes also resulting in an increased (7.96% ± 4.35) nanoparticle uptake (Fig. 5a,b).

The observation that hypoxic conditioned MDA-MB-231 and 1833 cells showed consistently increased nanoparticle uptake raised the possibility that this response was due to (i) increased endocytosis (i.e. uptake), (ii) reduced exocytosis (i.e. recycling) or (iii) a combination of both (i) and (ii). This question was addressed by performing pulse chase experiments with normoxic and hypoxic conditioned cells. These studies were conducted with MDA-MB-231 and 1833 cells conditioned for 24 hours to hypoxia and pulse dosed for 45 minutes, as this treatment gave the greatest relative increase in nanoparticle uptake (Figs 4c and 5c). After this treatment, the cells were chased for 30 minutes and then analysed. Comparison of the baseline cell-associated fluorescence with post chase cell-associated fluorescence demonstrated a post-chase drop in the MDA-MB-231 cells which was significantly greater following hypoxic conditioning than following normoxic conditioning (81.13% ± 2.18, *n* = 15 and 72.14% ± 4.94, *n* = 15, respectively) (Fig. 4). Similarly the post-chase drop in the 1833 cells was also significantly greater following hypoxic conditioning than with normoxic conditioning (52.09% ± 4.75, *n* = 10 and 43.74% ± 10.13, *n* = 10, respectively) (Fig. 5c).

## Discussion

We believe this is the first quantitative study on human breast cancer cells that assesses the impact of hypoxic adaptation on both nanomedicine internalisation and recycling whilst also taking into account the temporal changes in key elements of the hypoxic adaptation circuit itself. Recent mechanistic insights into nanomedicine access to tumor cells includes transient vascular bursts^41,42^ and a combination of radiotherapy and tumor associated macrophages (TAM) that show the potential to enhance therapeutic delivery of nanomedicines via the characteristically short periodic vascular reperfusion^43^ found in tumors. In experimental xenografts these vascular burst were dependent on blood flow, were intermittent and facilitated nanomedicine distribution into large areas of the tumor (100s μ^2^) (ref.^42^). We therefore designed our uptake studies so that nanomedicine dosing occurred at the start of short periods of re-oxygenation, followed by hypoxia, to reflect the current understanding of transient tumor vascular reperfusion^44^, and the subsequent access of nanomedicines to the intratumoral space.

This study examined the impact of hypoxia on nanoparticle endo- and exocytosis using a simple, but yet effective and well-controlled two-dimensional *in vitro* culture system. The merit of our system is the ability to both monitor pericellular oxygen levels non-invasively and to quantify nanoparticle uptake and efflux. Unlike three-dimensional organotypic culture models our model system reduced the complexity thus eliminating co-founding factors such as mass transport limitations (of both nanoparticles and oxygen) and permitting rapid sample processing and analysis. Rapid sample handling is important to ensure that endocytosis and exocytosis is arrested (here by placing samples on ice). While this study demonstrates the basic role of hypoxia in nanoparticle uptake and efflux, many other factors are likely to impact nanomedicine performance. A number of normoxic studies have considered factors like stability of the carrier, cargo release and particle size and elasticity^45^. However, this study was deigned specifically to exclude as many of these confounding factors as possible.

Our data showed that nanomedicine internalisation is altered in a dynamic fashion in response to varying periods of hypoxic conditioning and dosing intervals. These findings paralleled the altered expression of key proteins within the hypoxic adaptation circuit itself. MDA-MB-231 cells increased their capacity for internalisation of nanoparticles in response to hypoxic conditioning for 6 or 24 hours, with the greatest difference observed following 24 hours hypoxia. In addition, following all hypoxic conditioning periods, the greatest relative increase in internalisation was observed over a 45 minute dosing interval. The MDA-MB-231 parent cell line is heterogeneous and contains adapted, highly metastatic sub-populations^40,46^. We therefore examined the performance of nanoparticles in the bone metastatic subline 1833 because bone metastasis is common in triple negative breast cancer^47^. We examined the cellular response in MDA-MB-231 and 1833 cells (classified as mesenchymal-like) to make inroads into the effects of tumor heterogeneity (within the same patient) on nanomedicine uptake and efflux in hypoxia. However, gene expression analysis of triple negative breast cancer has identified six main subtypes (mesenchymal-like cells are one of them)^48^. It thus remains to be seen how all these different subclasses of triple negative cells respond to hypoxia.

Similarly to the parent cell line, 24 hour hypoxic conditioning of 1833 cells followed by a 45 minute dosing interval showed a significant increase in nanoparticle uptake. Furthermore, both the MDA-MB-231 and 1833 cell lines conditioned to hypoxia for 24 hours increased their exocytosis of nanoparticles. We therefore speculate that hypoxic endocytic uptake and recycling are similar in both these mesenchymal-like breast cancer cell lines. However, systematic studies examining these differences have not been reported. Overall, these results demonstrate that intratumoral hypoxia has the potential to alter nanomedicine uptake by tumor cells, thereby modifying intracellular trafficking and confounding effective therapeutic payload delivery.

A key aspect of the hypoxic response is the shift from energy-efficient oxidative phosphorylation to the less productive, yet oxygen conserving, glycolytic pathway^49–51^. As a consequence, hypoxic tumor cells enter a lower energy state associated with reduced ATP synthesis. Because nanomedicine internalisation via endocytosis is an active, energy-dependent process, the expectation would be a reduction in nanomedicine internalisation, yet here, we observed a significant increase. There are however, few studies available for comparison with our work. One study reported a reduction in the cellular uptake of 1.9 nm gold nanoparticles in MDA-MB-231 cells under hypoxic conditions^52^. However, that study employed a very low (0.1%) oxygen environment for hypoxic conditioning and the conditioning was only for 4 hours prior to dosing with nanoparticles. Some cellular hypoxic adaptations would be expected over that short duration of hypoxia, but no validations of molecular changes were reported and pericellular oxygen levels were not determined. In the present study, therefore, we adopted *in situ* pericellular oxygen monitoring and tracked 11 stress related proteins (including the master hypoxic effector, HIF1α) as markers of hypoxic cellular conditioning and the cellular hypoxic response, respectively. By contrast, Neshatian *et al*.^53^ demonstrated elevated internalisation of gold nanoparticles (sizes: 15, 50 and 70 nm) in human MCF-7 breast cancer cells following 18 hours of hypoxic pre-conditioning in a very low 0.2% oxygen environment. For our study, we selected a hypoxic oxygen level of 1% because meta-analysis^54^ of *in vivo* ultrasound guided hypoxia measurements within human breast tumors indicated that the median pO_2_ was 10 mmHg. Using normobaric assumptions, this approximates to around 1.0 to 1.3% oxygen. As expected, we found lower pericellular oxygen in the presence of cells than in cell-free media (Fig. 1). This suggests that an incubation environment as low as 0.2 or 0.1% O_2_, as used in the previous published studies, could lead to anoxic, as opposed to hypoxic, conditions within the cells themselves^55^. This is important, because anoxia is known to trigger alternative cellular responses (e.g. activating transcription factor 3 and 4) that are not mediated via HIF^56,57^. This raises concerns about the relevance of these previously published studies in the context of nanomedicines and limits our ability to compare our work with them.

Overall, nanomedicine retention within cells is the sum of both uptake (i.e. endocytosis) and efflux (i.e. exocytosis)^3^ and requires a mechanism for regulation of cellular homeostasis (e.g. cell volume, plasma membrane economics)^58^ and for response to and modulation of cell signalling (e.g. receptor recycling versus down regulation). Therefore, assessment of the endocytic index of nanomedicines must include both endocytosis and exocytosis^3^. The current results showed that 24 hour hypoxic preconditioning increased nanomedicine uptake, but it also increased exocytosis (Figs 4 and 5). The degree and speed of recycling under normoxic conditions we have observed here is similar to that noted in previous work (albeit, under differing experimental conditions)^59,60^.

The observed upregulation of the energy dependent processes of endo- and exocytosis, in what is ostensibly a low energy hypoxic cellular state, would appear to be counterintuitive. However, tumor cell hypoxic adaptation involves well-established changes to endocytic receptor uptake and signalling^35,61,62^ and altered intracellular trafficking^36^. Recent *in vitro* research has shown that MDA-MB-231 and HeLa cells undergo a generalised reduction in overall internalisation of the tumor cell surface proteome in response to hypoxia, with a parallel selective upregulation of specific endocytic pathways, mediated via caveolin 1 (ref.^63^). Further, the recycling of transmembrane proteins may also be influenced by interaction with proteins like Caveolin 1, among others^64^. Interestingly, constitutive *in vitro* expression of Caveolin 1 is markedly higher in MDA-MB-231 cells than in many other tumor cell lines^65,66^, suggesting its potential for a greater influence in this cell line. Similarly, upregulation of exocytotic release of exosomes or vesicles from tumor cells during hypoxia is known to play a significant role in tumor development and signaling^37,67^, with implications for altered or upregulated exocytosis. Thus, our results may reflect these types of specific upregulated endocytic and exocytic processes, which deserve further investigation.

Our study measured relative expression of HIF1α, the master effector of hypoxic adaptation, to assess how the cellular hypoxic response might change with the duration of hypoxic exposure. Unhydroxylated HIF1α expression was increased approximately four fold when compared to normoxic levels after 6 hours of hypoxia, but returned to near normoxic levels following 24 hours of hypoxia. Similar temporal patterns of HIF1α expression have been demonstrated in MDA-MB-231 cells exposed to similar *in vitro* hypoxic conditions^68^. The regulation of this cyclical HIF1α expression is multifactorial, but it appears to be driven principally by a variety of cellular factors, including REST (repressor element 1- silencing transcription factor)^68^. In the context of our results, it is interesting to note that we observed the greatest differences in nanoparticle internalisation following 24 hours of hypoxic conditioning, where we also found that HIF1α had returned to near normal levels.

We assessed the relative expression of ten other key cell stress proteins in the MDA-MB-231 cells and demonstrated that their altered expression depended on the duration of hypoxia. Whilst all these proteins are relevant to the cellular stress response to hypoxia, of particular note are those known to form part of the HIF1 regulatory circuit. For example, CbP/p300 – interacting transactivator – 2 (Cited 2) is a known HIF1 negative regulatory element that exhibits preferential binding of CBP/p300 co-factors required for HIF1 transcriptional activity^69^. Similarly, the NAD dependent deacetylase Sirtuin2 (SIRT2) has been shown, through deacetylation, to increase proteasomal breakdown of HIF1 via enhanced affinity for PHD2^70^. We found that the relative expression of Cited 2 peaked following six hours of hypoxia, whereas Sirtuin2 exhibited the highest relative expression following 24 hours of hypoxic conditioning. Taken within the context of our studies, these results underline the dynamic nature of hypoxic adaptation within tumor cells and its impact on the uptake and efflux of nanomedicines during hypoxia.

## Conclusions

**Figure 6:**
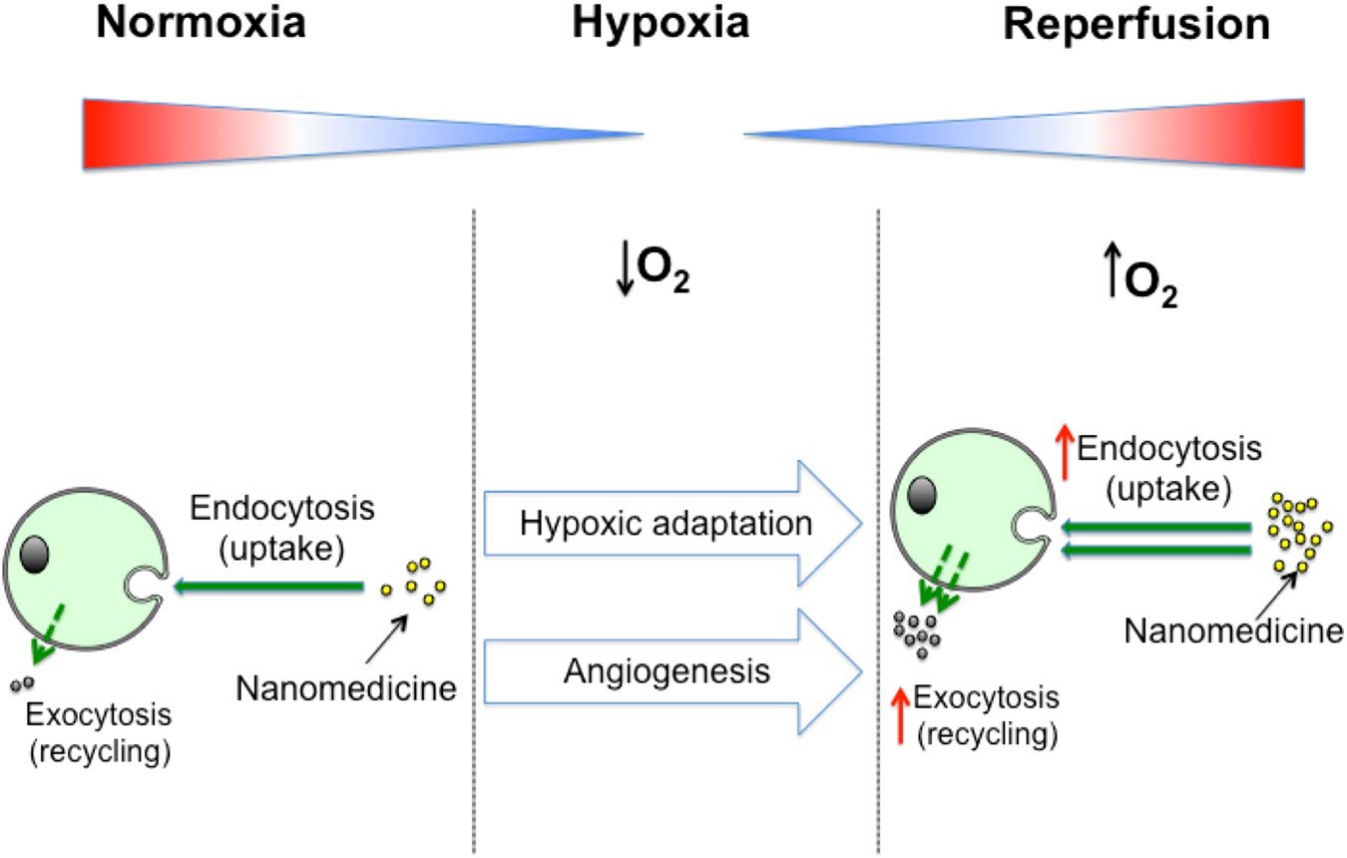
A schematic summary of key findings: Hypoxic conditioning of breast cancer cells increases nanomedicine uptake and efflux.

The objective of this work was to quantify the difference that tumor cell hypoxic adaptation might make to *in vitro* nanomedicine uptake and recycling (Fig. 6). We demonstrated that both uptake and recycling of our model nanomedicine were increased following hypoxic incubation in both the MDA-MB-231 and 1833 cell line. Further, we demonstrated with the MDA-MB-231 cell line, that the magnitude of these changes depended on the duration of the hypoxic exposure and the dosing interval. Overall, these results expand the existing knowledge of how the hypoxic tumor microenvironment can potentially alter nanomedicine internalisation, with implications for effective therapeutic delivery and design.

## Experimental Section

### Cell culture

MDA-MB-231 cells (ATCC^®^ HTB-26™) were purchased from the American Type Culture Collection (Manassas, VA, USA). The 1833 subline was gifted by Dr. Joan Massagué (Memorial Sloan-Kettering Cancer Center, New York, NY, USA) and detailed elsewhere^40^. Cells were cultured as monolayers in RPMI 1640 media (Life Technologies, UK), supplemented with 10% (v/v) foetal bovine serum (FBS), 50 U/mL penicillin and 50 μg/mL streptomycin. Unless otherwise indicated, cells were seeded at 4 × 10^4^ cells/cm^2^. For all experimental work, hypoxic or normoxic culture conditions were achieved using a gas mixture of 5% CO_2_, 1% O_2_ and 87.8% N_2_ (hypoxic) or 5% CO_2_, 18.6% O_2_ and 70.2% N_2_ (normoxic) within a humid 37°C incubator. Normobaric conditions were assumed throughout.

### Fluorescent nanoparticles

Fluoresbrite® spherical fluorescent polystyrene nanoparticles (diameter 43.4 nm ± 4.4 with an excitation/emission maxima at 441/486 nm), suspended in water, were purchased from Polysciences Europe GmbH, Eppelheim, Germany.

### *In vitro* cytotoxicity studies

MDA-MB-231 cells were seeded into 96-well tissue culture treated polystyrene plates (TPP Techno Plastic Products AG, Trasadingen, Switzerland) at a density of 3 × 10^3^ cells/cm^2^ in 100 μl complete culture medium. The plates were then incubated in either normoxic or hypoxic conditions for 24 hours. The wells were then aspirated and fresh media containing nanoparticles at a range of concentrations up to 1 × 10^11^ nanoparticles/ml was added, followed by a further incubation in the respective environment for 44 hours. Next, 20 μl of (3-(4,5- dimethylthiazol-2-yl)-2,5-diphenyltetrazolium bromide (MTT; 5 mg/ml in PBS) was added and incubated for 4 hours. The MTT was aspirated from all wells and formazan crystals were dissolved in dimethyl sulphoxide and absorbance read at 570 nm. Cell viability at each nanoparticle dose was calculated as a percentage of the control (i.e. zero dose).

### *In vitro* trafficking of fluorescent nanoparticles

MDA-MB-231 cells were seeded onto cell culture treated polystyrene Cellstar® cell culture dishes (Greiner Bio-One, Kremsmunster, Austria) for 24 hours in either hypoxic or normoxic conditions (as defined above). Next, cells were dosed with fluorescent nanoparticles for either 45 or 180 minutes, placed on ice and washed twice with PBS at 4 °C, stored on ice and transferred for confocal imaging immediately. Lysosomal staining was achieved using LysoTracker® Red (Invitrogen, Waltham, MA, USA) according to the manufacturer’s instructions. Live cell confocal co-localisation imaging was conducted using a Leica TCS SP5 laser scanning confocal microscope equipped with a 40× liquid immersion objective. Confocal slices were assembled into figures, brightness/contrast adjusted using ImageJ v1.0 (National Institutes of Health, Bethesda, Maryland, USA) and imported into Graphpad Prism® v7.0 (GraphPad Software Inc., La Jolla, CA, USA).

### Generation of cell lysates

MDA-MB-231 cells were seeded in 75 cm^2^ tissue culture treated polystyrene culture flasks and incubated for 24 hours under normoxic conditions to support cell growth. Next, flasks were split into 4 groups and cultured for a further 24 hours using specific conditioning regimes: (i) normoxic control, (ii) 6 hours of hypoxia (i.e. 18 hours normoxia followed by 6 hours of hypoxia conditioning), (iii) 24 hours of hypoxic conditioning and (iv) positive control using 100 μM CoCl_2_. The CoCl_2_ dosing served to chemically block HIF1α breakdown in the presence of oxygen. At the end of the conditioning regime, samples were immediately immersed in ice. Within 90 seconds, the culture medium was removed and the cell monolayers were washed twice with 5 ml ice cold PBS, followed by 1.0 ml of ice cold radioimmunoprecipitation (RIPA) buffer containing 40 μl of 25 × Roche Diagnostics Easypack® Protease cocktail (both from Sigma-Aldrich, Dorset, England, UK). The cells were then harvested using a cell scraper. The lysates were pipetted into ice cold centrifuge tubes and, while maintained at 4 °C, vortexed at full power for 1 minute, shaken at full power for 20 minutes and then centrifuged for 20 minutes at 12,000 × g. Following centrifugation, the supernatant was aliquoted and stored at −80°C until further analysis.

### Protein separation, western blotting and protein arrays

The total protein concentration of each cell lysate was determined using the Pierce™ bicinchoninic acid protein assay kit (Thermo Scientific, Waltham, MA, USA). Protein samples were denatured in a 1:1 ratio using Laemmli sample loading buffer [65.8 mM Tris-HCl, pH 6.8, 2.1% (w/v) SDS, 26.3% (w/v) glycerol, 0.01% (v/v) bromophenol blue, and 5% (v/v) 14.2 M β-mercaptoethanol (Bio-Rad Laboratories, Hemel Hempstead, UK)] by heating for 5 minutes at 95 °C. Equivalent protein quantities (25 μg) and a Precision Plus Kaleidoscope™ protein ladder (Bio-Rad Laboratories, Hemel Hempstead, UK) were loaded on to an 8% polyacrylamide gel, and separated at 150 V for 55 minutes. Proteins were blotted onto a polyvinylidene difluoride membrane (Bio-Rad Laboratories, Hemel Hempstead, UK). The following antibodies were used to probe the membrane: Rabbit primary antibodies for β-actin (1:10,000) and unhydroxylated HIF1-α (1:1,000) (monoclonal and polyclonal respectively) as well as monoclonal goat anti-rabbit IgG HRP linked secondary antibody (1:2,000) (all from Cell Signalling Technology, Danvers, MA, USA). Primary antibodies were blocked with 5%w/v bovine serum albumin (Sigma-Aldrich, Dorset, England, UK) in tris-buffered saline Tween (TBST), with secondary antibody blocking achieved with 5% non-fat dry milk powder in TBST. Relevant bands were visualised using Clarity™western ECL substrate and UltraCruz autoradiography film (Santa Cruz Biotech Inc., Dallas, TX, USA). HIF1-α and β-actin bands were digitised (Epson Perfection v600 film flatbed scanner, Epson Europe, B.V., Netherlands), and densitometry scans were completed using Image Studio™ Lite software (LI-COR Biotechnology, Lincoln, NE, USA). The relative expression of 11 cell stress-associated proteins was determined using the R&D Proteome profiler™ (catalogue number #ARY018; R&D Systems, Inc., MN, USA), following the manufacturer’s instructions. Triplicates of cell lysates were prepared as detailed above and pooled, with a total of 300 μg protein per sample used. Arrays were imaged and analysed by densitometry, as detailed above.

### Normoxic and hypoxic cell cultures and nanoparticle uptake and release

MDA-MB-231 or 1833 cells were seeded into 6-well plates. Plates were then incubated for either (i) 24 hours in hypoxic conditions, (ii) 24 hours in normoxic conditions, or (iii) 18 hours under normoxia followed by 6 hours under hypoxia. Cells were then dosed with nanoparticles at an effective concentration of 1 × 10^10^ nanoparticles/ml (1 × 10^11^ nanoparticles/ml for 1833 cells), and the plates were returned to their respective culture environments for either 45 or 180 minutes. For the recycling studies, cells were preconditioned for 24 hours in either hypoxia (1% O_2_) or normoxia. Next, cells were dosed with nanoparticles at an effective concentration of 1 × 10^10^ nanoparticles/ml (1 × 10^11^ nanoparticles/ml for 1833 cells) for 45 minutes and analysed (baseline), or washed and allowed to exocytose for 30 minutes (exocytosis). Within the next 15 minute time interval, the cells were washed 3 times with ice cold PBS, detached using trypsin and transferred to flow cytometry tubes for analysis. Flow cytometry was performed using a FACS Canto™II FACS analyser (Becton Dickinson, Oxford, England, UK) by assessing mean cell-associated fluorescence with an argon laser (excitation 488 nm, emission 525 nm) and gating 10,000 events. For all hypoxic measurements normoxic control groups were run in parallel. Mean FITC fluorescence values were determined from FCS 3.0 files using FlowJo® v10.3 software (FlowJo LLC, Oregon, USA). Mean FITC fluorescence values (Supplementary Fig. S4) were converted into percentages to calculate relative differences.

### Pericellular oxygen monitoring

Three wells of Presens Oxohydrodish® 6 well plates (Presens, Precision Sensing GmbH, Regensburg, Germany) were seeded with MDA-MB-231 cells, as described above. The remaining three wells were filled with an equivalent volume of medium only. Each plate was then placed on the Presens Sensordish® 24-channel plate reader within the hypoxic incubator. Following temperature equilibration, the in-well oxygen percentage was recorded for each well, separately, at frequent intervals, for a period of up to 26 hours.

### Statistical analyses

Statistical analyses were performed using GraphPad Prism® v6.0 (Graphpad Software Inc., La Jolla, CA, USA). All significance tests used unpaired two tailed Student’s *t* tests, except for cytotoxicity measurements, where a one-way unpaired ANOVA with Sidak multiple comparisons test was used (α = 0.05). Asterisks denote statistical significance as follows: *p < 0.05, **p < 0.01 and ***p < 0.001. All data are presented as a mean values ± standard deviation (SD), unless otherwise stated.

## Electronic supplementary material

Supplementary Information

## Data Availability

All data created during this research are openly available from the University of Strathclyde-Pure, at 10.15129/3b637e0e-6b92-4041-bac9-0ed9bc057bfd.
