# Supplementary material for: Impact of the hypoxic phenotype on the uptake and efflux of nanoparticles by human breast cancer cells
Source: Sci Rep. 2018 Aug 17;8:12318. doi: 10.1038/s41598-018-30517-3 (PMC6098061; doi:10.1038/s41598-018-30517-3)
Supplement: Supplementary file 1 — Supplementary Information [file 41598_2018_30517_MOESM1_ESM.doc]

Impact of the hypoxic phenotype on the uptake and efflux of nanoparticles by human breast cancer cells

*William J. Brownlee^#^, F. Philipp Seib ^#+*^*

^#^ Strathclyde Institute of Pharmacy and Biomedical Sciences, University of Strathclyde, 161 Cathedral Street, Glasgow, G4 0RE, UK. E-mail: philipp.seib@strath.ac.uk

^+^ Leibniz Institute of Polymer Research Dresden, Max Bergmann Center of Biomaterials Dresden, Hohe Strasse 6, 01069 Dresden, Germany

* Corresponding author: Tel. +44 (0) 141 548 2510, E-mail: philipp.seib@strath.ac.uk or philipp.seib@SeibLab.com

**Supplementary information**


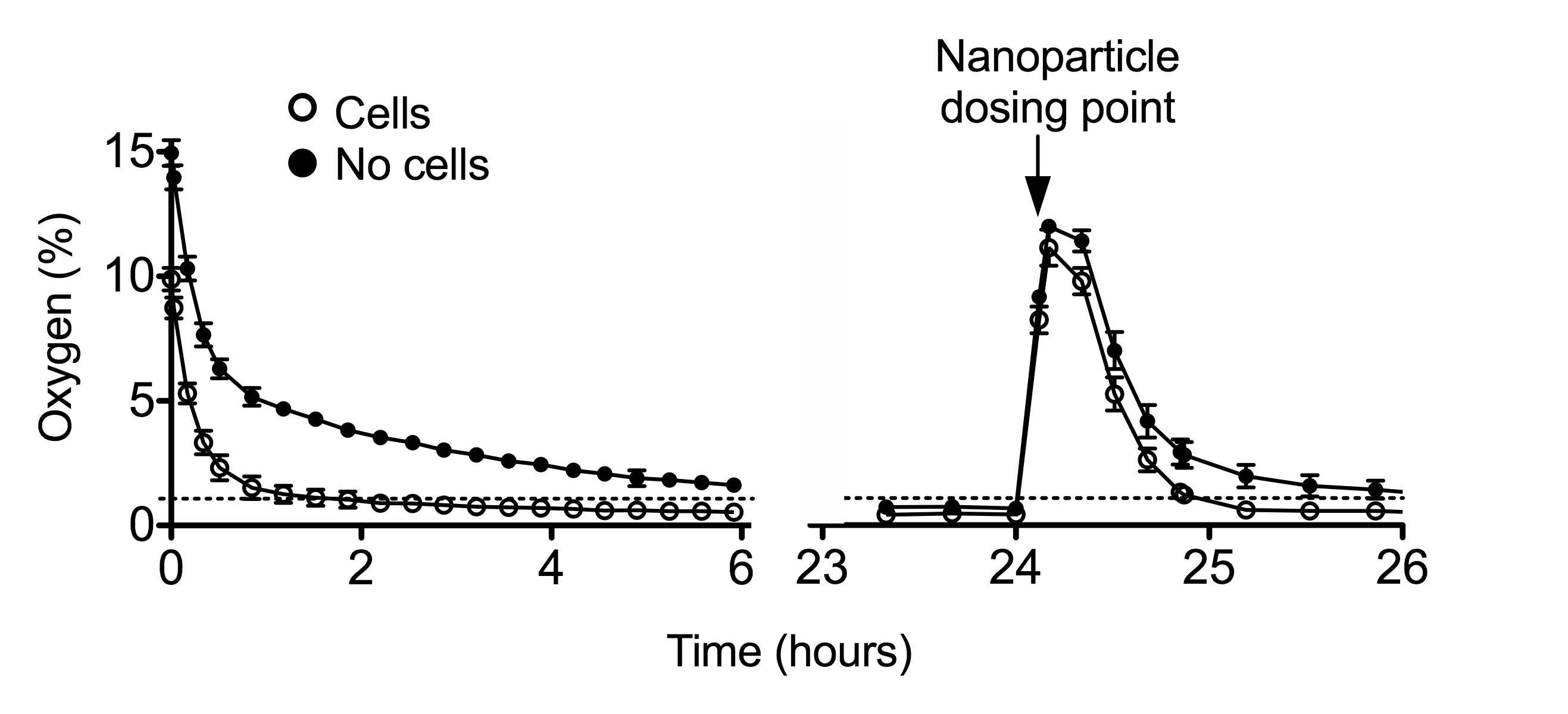


**Supplementary Figure S1.** Pericellular oxygen monitoring. (a) Magnification of the first 6 hours of culture and (b) oxygen profile during a nanoparticle dosing point emulating re-oxygenation. Data average n=3 ± SD.


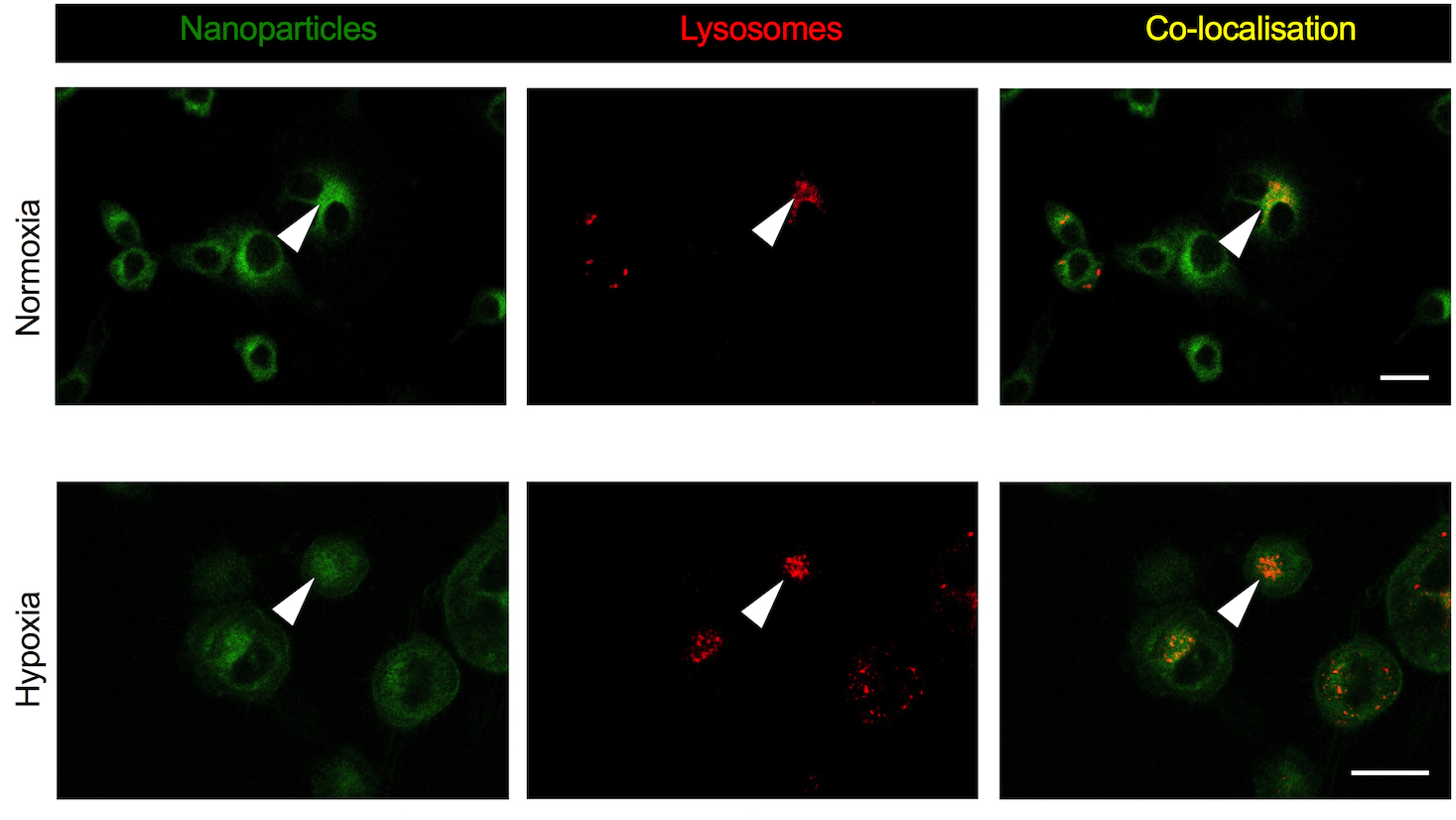


**Supplementary Figure S2.** uptake of nanoparticles in response to normoxia and hypoxia. Live cell confocal imaging of cells exposed for 24 hours to normoxia or hypoxia and subsequently dosed for 180 minutes with nanoparticles (green). Acidic vesicles were stained using LysoTracker Red. Arrows show nanoparticle co-localisation in acidic vesicles. Scale bar 20 μm


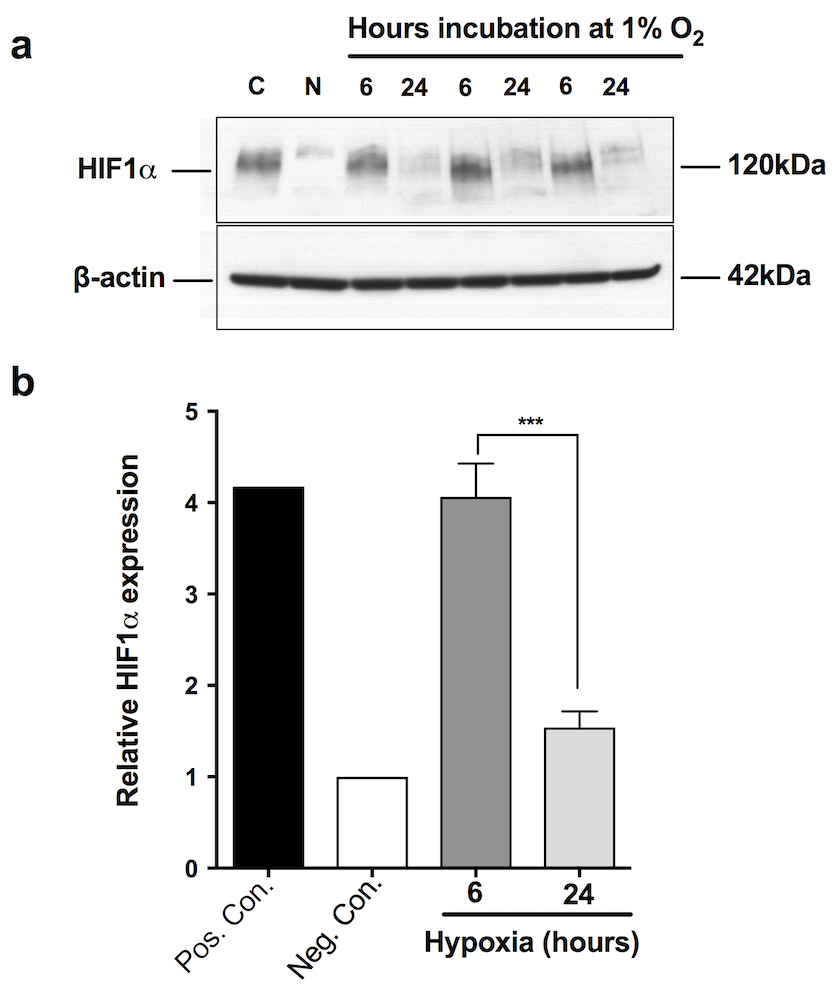


**Supplementary Figure S3.**  SDS PAGE and immunoblotting of cell lysates. (**a**) Blots from 3 independent experiments, stained for unhydroxylated HIF1α (β-actin is loading control); C: positive control; N: normoxic control. (**b**) Densitometry of (a), expressed as fold change relative to normoxia (i.e. set to 1.0); ± SD, *n* = 3 from independent biological experiments. Positive control (Pos Con) cells were treated with 100 μM CoCl_2_.

**
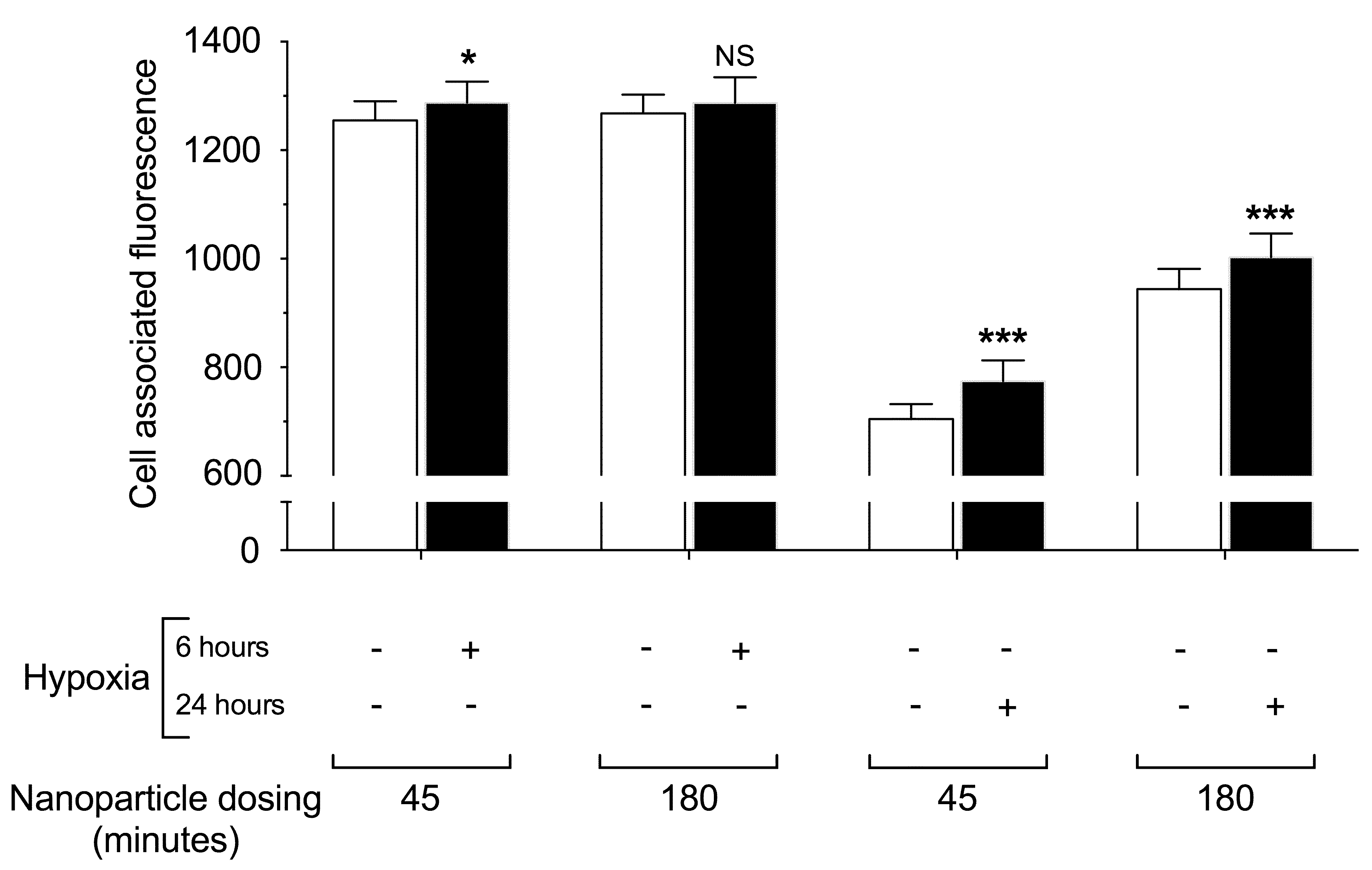
**

**Supplementary Figure S4.** Impact of hypoxic preconditioning on the uptake of nanoparticles by human MDA-MB-231 breast cancer cells. Raw data set accompanying Figure 4b. Cells were conditioned in hypoxia (1% O2) for either 6 or 24 hours and then dosed with nanoparticles for either 45 or 180 minutes. Uptake of fluorescent nanoparticles was assessed by measuring mean single cell-associated fluorescence by flow cytometry; ≥10,000 events and n = 15, per treatment group and dosing interval, from 3 independent biological experiments ± SD.
